# N6-Methyladenosine RNA Methylation Regulators Have Clinical Prognostic Values in Hepatocellular Carcinoma

**DOI:** 10.3389/fgene.2020.00863

**Published:** 2020-08-12

**Authors:** Wei Liu, Cuiqing Zhong, Deguan Lv, Mengjie Tang, Feng Xie

**Affiliations:** ^1^Department of Pharmacy, The Third Xiangya Hospital, Central South University, Changsha, China; ^2^Center for Reproductive Medicine, Ren Ji Hospital, School of Medicine, Shanghai Jiao Tong University, Shanghai, China; ^3^Division of Regenerative Medicine, Department of Medicine, University of California, San Diego, San Diego, CA, United States; ^4^Hunan Cancer Hospital, The Affiliated Cancer Hospital of Xiangya School of Medicine, Central South University, Changsha, China; ^5^Department of Pharmacy, The Nanshan District Maternity and Child Healthcare Hospital of Shenzhen, Shenzhen, China

**Keywords:** m^6^A modification, m^6^A regulators, hepatocellular carcinoma, a risk signature, prognostic marker

## Abstract

Although it is widely accepted that N6-methyladenosine (m^6^A) RNA methylation plays critical roles in tumorigenesis and progression, the values of m^6^A modification are less known in hepatocellular carcinoma. The major purpose of our current studies is to investigate the role of m^6^A regulators in hepatocellular carcinoma and whether it can affect the prognosis of hepatocellular carcinoma. Here we demonstrate that most of the m^6^A regulators are highly expressed in hepatocellular carcinoma. Furthermore, we cluster hepatocellular carcinoma into two subgroups (cluster 1/2) by applying consensus clustering to m^6^A regulators. Compared with the cluster 1 subgroup, the cluster 2 subgroup was significantly associated with a higher pathological grade and survival. Based on these findings, we reveal a risk signature by using three m^6^A regulators, which are not only an independent prognostic marker but also a predictor of the clinicopathological features in hepatocellular carcinoma. In conclusion, m^6^A regulators are crucial participants in the malignant progression of hepatocellular carcinoma and are potential targets for prognosis.

## Introduction

RNA modification was first discovered in the 1960s and was considered to be another epigenetic form analogous to DNA and histone modification (Jia et al., 2013). Among more than 100 kinds of RNA modifications known so far, N6-methyladenosine (m^6^A) methylation is the most abundant RNA epigenetic modification in RNA, which is dynamically regulated by methyltransferases (“writers”), binding proteins (“readers”), and demethylases (“erasers”) (Niu et al., 2013; Yang et al., 2018). The prominent methyltransferases complex catalyzes the formation of m^6^A, which contain at least six “writer” proteins: methyltransferase like 3 (METTL3), methyltransferase like 14 (METTL14), WT1-associated protein (WTAP), VIRMA (KIAA1429), zinc finger CCCH domain-containing protein 13 (ZC3H13), and RNA binding motif protein 15 (RBM15) (Liu and Pan, 2016). The demethylases catalyze the demethylation of m^6^A, which mainly include fat mass- and obesity-associated protein (FTO) and α-ketoglutarate-dependent dioxygenase alkB homolog 5 (ALKBH5) (Ding et al., 2018; Piette and Moore, 2018). The binding proteins, which recognize and bind with m^6^A, are consisting of YTH domain family proteins and heterogeneous nuclear ribonucleoprotein C (HNRNPC) (Duan et al., 2019). The biological functions of m^6^A RNA methylation are involved in regulating all stages of the RNA life cycle, including pre-mRNA splicing, pri-miRNA processing, nuclear output, RNA translation regulation, and RNA degradation (Roignant and Soller, 2017).

The transcriptome-wide mapping of m^6^A focuses on investigating the landscapes and the functions of the reversible m^6^A modification in the last decade (Bi et al., 2019). Recently, more and more scientists focus on exploring the association between m^6^A and human diseases, especially in tumors (Dai et al., 2018; Pan et al., 2018). A growing appreciation of the biological significance of m^6^A RNA methylation implied that m^6^A contributed to tumorigenesis and tumor progression (Deng et al., 2018). The dislocation of m^6^A is closely associated with various kinds of cancers, such as glioblastoma (GBM), colorectal carcinoma (CRC), pancreatic cancer (PC), and hepatocellular carcinoma (HCC) (Chen et al., 2018; Chai et al., 2019; Zhang et al., 2019; Zhou et al., 2019). Notably, the roles of m^6^A regulators in tumors are controversial. METTL3 serves as a tumor suppressor gene in GBM and is considered as an oncogene in CRC or non-small cell lung carcinoma (Li et al., 2019; Wei et al., 2019; Liu et al., 2020). YTHDF2 acts as a tumor suppressor gene in lung cancer and supposed to be an oncogene in PC (Chen et al., 2017; Sheng et al., 2019). The controversial roles of m^6^A regulators in tumors suggest that the functions of m^6^A modification in tumors are complicated. Moreover, the literature does not have comprehensive m^6^A regulator expression and prognosis analysis in tumors.

In this study, we systematically analyze the expression data of 13 m^6^A modification regulators in HCC from The Cancer Genome Atlas (TCGA) datasets. We demonstrate that most of the 13 m^6^A regulators are highly expressed among HCC. Moreover, we also find that the m^6^A regulators are crucial participants in the malignant progression of HCC and a signature with three selected m^6^A regulators is designed to stratify the prognosis of HCC.

## Materials and Methods

### Data Acquisition and Processing

**FIGURE 1:**
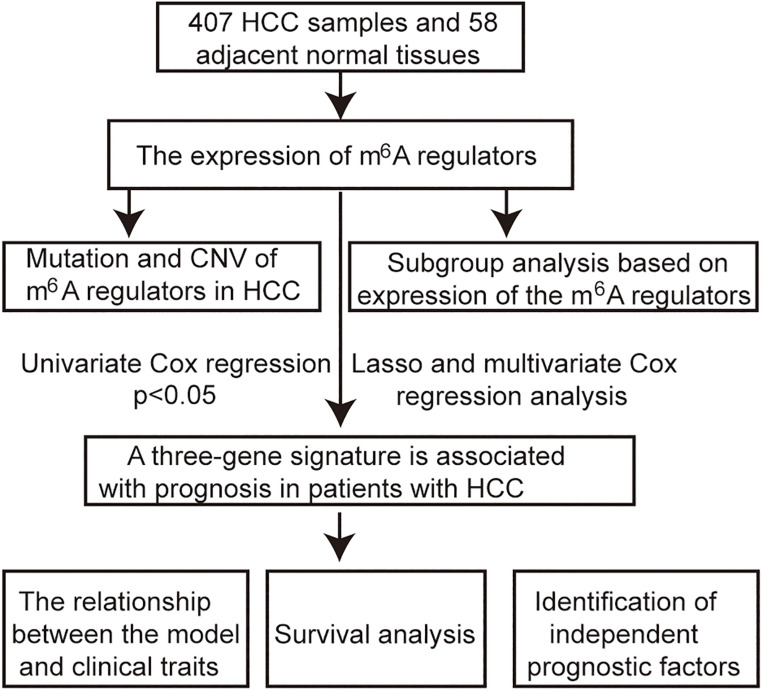
Flow chart of the approach utilized in the current study.

The RNA-seq transcriptome and clinical data of 407 HCC samples and 58 adjacent tissue samples were obtained from TCGA^1^. The workflow type is fragments per kilobase million. The R package “limma” was used to process and delete duplicate genes. The expression of m^6^A regulators in HCC was extracted from RNA-seq transcriptome. The Wilcoxon test was used to analyze the differential expression of these m^6^A regulators (*p* < 0.05 was considered as significant). Incomplete samples of survival data were removed, and finally, 403 samples with complete clinical information were obtained for subsequent analysis. The flow chart of this study is shown in Figure 1.

### Identify the Role of m6A Regulators in HCC

Gene mutation and copy number variation data were downloaded from the cbioport database^2^. The interaction and the correlation among m^6^A regulators were analyzed using the R package “corrplot.” The HCC patients were divided into two subgroups based on the expression of m^6^A regulators using a cluster analysis method with “ConsensusClusterPlus”^3^. The R package “ggplot2” is used for principal component analysis (PCA). The R package “survival” was used to plot Kaplan–Meier survival curves. A *p* < 0.05 was considered as statistically significant.

### Construction of a Signature Associated With Prognosis

The roles of m^6^A regulators in the prognosis of HCC patients were identified by univariate Cox regression analysis; *p* < 0.05 was considered as significant. A risk signature was built by the least absolute shrinkage and selection operator (LASSO) Cox regression algorithm, and multivariate Cox regression analysis. The signature is expressed as follows: risk score = (coefficient gene 1 × gene 1 expression) + (coefficient gene 2 × expression of gene 2) + … + (coefficient gene *n* × expression gene *n*). The median risk score served as a cutoff value to classify patients into high-risk and low-risk groups. The R package “survival ROC” was used to perform time-dependent receiver operating characteristic (ROC) curve analysis to assess the accuracy of the predicted genetic features of time-dependent cancer death. The area under the curve (AUC) was calculated to evaluate the accuracy of the risk prediction model. The R package “survival” was used to plot Kaplan–Meier survival curves.

### Independence of Prognostic Factors From Other Clinical Parameters in TCGA

Complete information on the 403 samples included relevant clinical data for univariate and multivariate Cox regression analyses. *p* < 0.05 was considered as statistically significant.

### Construction of a Predictive Nomogram

The independent prognostic factors were chosen as the prognostic model to construct a nomogram in the entire TCGA cohort. The calibration plot and the concordance index (C-index) were used to investigate the calibration and the discrimination of the nomogram.

## Results

### m^6^A Regulators in HCC Patients Are Highly Expressed

**FIGURE 2:**
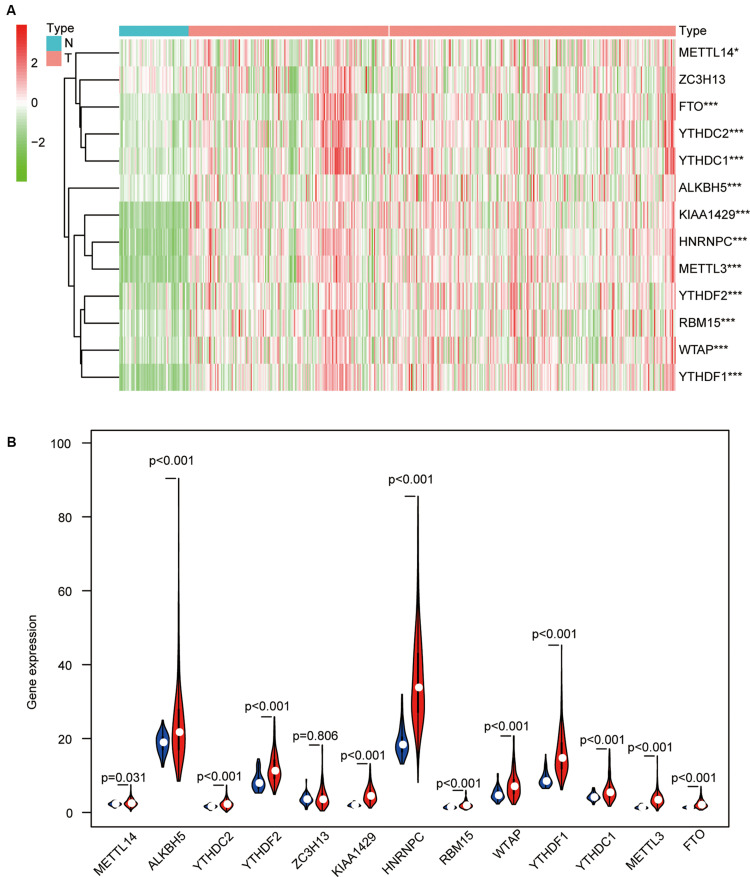
The differential expression levels of 13 m^6^A regulators in hepatocellular carcinoma (HCC) tissues and adjacent normal tissues. **(A)** The results of the heat map show the expression levels of 13 m^6^A regulators in 407 HCC samples and 58 adjacent normal tissues (Wilcoxon test). **(B)** The histogram shows the differential expression levels of 13 m^6^A regulators in 407 HCC samples and 58 adjacent normal tissues (Wilcoxon test). *p < 0.05 and ***p < 0.001.

More and more reports have shown that m^6^A regulators such as METTL14 (Li et al., 2020), YTHDF1 (Zhao et al., 2018), YTHDF2 (Chen et al., 2018), and WTAP (Chen et al., 2019) are essential for the deterioration and the progression of HCC. To further confirm the role of all m^6^A regulators in HCC, we systematically investigated the expression of 13 m^6^A regulators (including six writers: KIAA1429, METTL3, METTL14, RBM15, WTAP, and ZC3H13; two erasers: ALKBH5 and FTO; and five readers: HNRNPC, YTHDC1, YTHDC2, YTHDF1, and YTHDF2) in 403 HCC samples and 58 adjacent normal tissue samples from the TCGA database. Information on these m^6^A regulators is shown in Table 1. Similar to the results of Li’s report (Li et al., 2020), we found that KIAA1429, METTL3, and HNRNPC are highly expressed in HCC tumor samples. Contrary to Li’s findings, our results show that the expression of METTL14, YTHDC1, YTHDC2, and FTO was also increased in HCC, while the expression of ZC3H13 has no difference between the tumor samples and the adjacent normal tissue samples. In detail, HNRNPC had the highest expression, followed by ALKBH5 and YTHDF1(*p* < 0.05) (Figures 2A,B). The inconsistent results between Li’s study and our research may be caused by different sample data.

**TABLE 1:** Information on 13 m^6^A regulators.

**Types**	**Gene symbol**	**HGNC symbol**	**Full name**
Readers	*HNRNPC*	5035	Heterogeneous nuclear ribonucleoprotein C
	*YTHDC1*	30626	YTH domain containing 1
	*YTHDC2*	24721	YTH domain containing 2
	*YTHDF1*	15867	YTH N6-methyladenosine RNA binding protein 1
	*YTHDF2*	31675	YTH N6-methyladenosine RNA binding protein 2
Writers	*KIAA1429*	24500	vir like m6A methyltransferase associated
	*METTL3*	17563	Methyltransferase like 3
	*METTL14*	29330	Methyltransferase like 14
	*RBM15*	14959	RNA binding motif protein 15
	*WTAP*	16846	WT1 associated protein
	*ZC3H13*	20368	Zinc finger CCCH-type containing 13
Erasers	*ALKBH5*	25996	alkB homolog 5, RNA demethylase
	*FTO*	24678	FTO alpha-ketoglutarate dependent dioxygenase

### Mutation and Copy Number Variation of m^6^A Regulatory Genes in HCC

**FIGURE 3:**
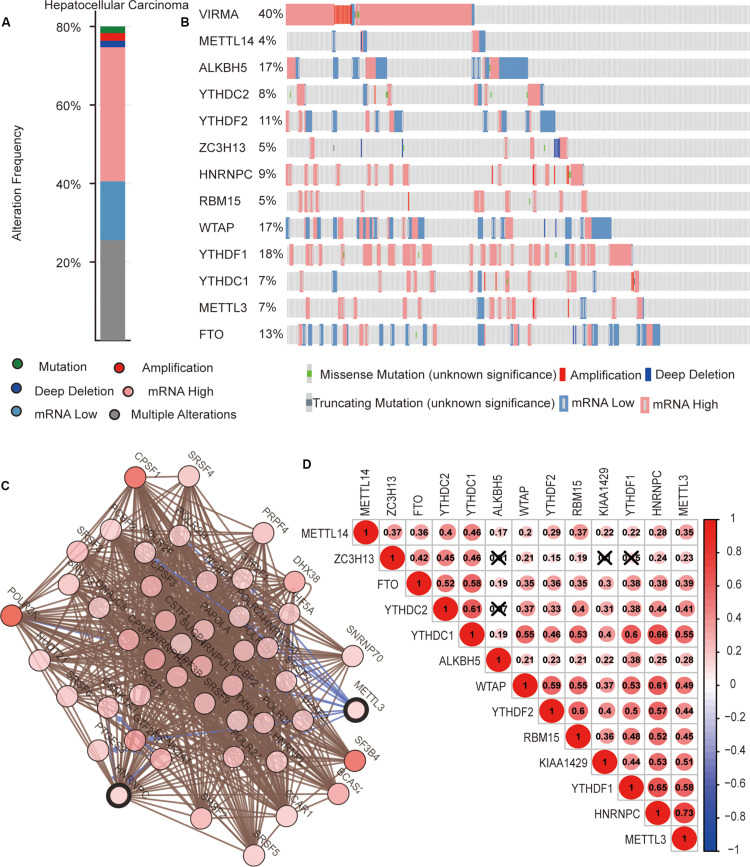
The interaction among m^6^A regulators in hepatocellular carcinoma (HCC). **(A,B)** The copy number variations and mutation of 13 m^6^A regulators in HCC from cbioport database. **(C)** The correlation of m6A regulator protein expression in HCC. **(D)** Correlation between the expression of 13 m6A regulators mRNA in HCC.

We then completely analyzed the different mutation and copy number variation (CNV) patterns of m6A regulatory genes in HCC from the cbioport database^4^. It included gene mutation, amplification, deep deletion, mRNA expression change, and other multiple alterations. The result revealed that m^6^A regulators were highly expressed in most HCC samples; meanwhile, m^6^A regulators had gene mutations and CNV (Figure 3A). Specifically, the m^6^A “writer” gene VIRMA (KIAA1429) had the highest mutation and CNV frequency (40%). as well as “readers” YTHDF1 (18%), ALKBH5 (17%), and WTAP (17%), respectively (Figure 3B).

### Interaction and Correlation Among m^6^A Regulators in HCC

Next, we evaluated the interaction and the correlation among m^6^A regulators. In the cbioport database, we found that there were close interactions among m^6^A regulators (Figure 3C). Furthermore, we analyzed the expression correlation of these genes in detail based on the expression profile of m^6^A regulators. The result showed that there was a significant positive correlation between the expressions of most m^6^A regulators. However, there might be no correlation between YTHDC2 and ALKBH5, ZC3H13 and ALKBH5, ZC3H13 and KIAA1429, and ZC3H13 and YTHDF1 (Figure 3D). These results reveal that, except for a few m^6^A regulators, most of them may play roles together in HCC.

### Classification of HCC Samples Based on the Expression of m^6^A Regulators

**FIGURE 4:**
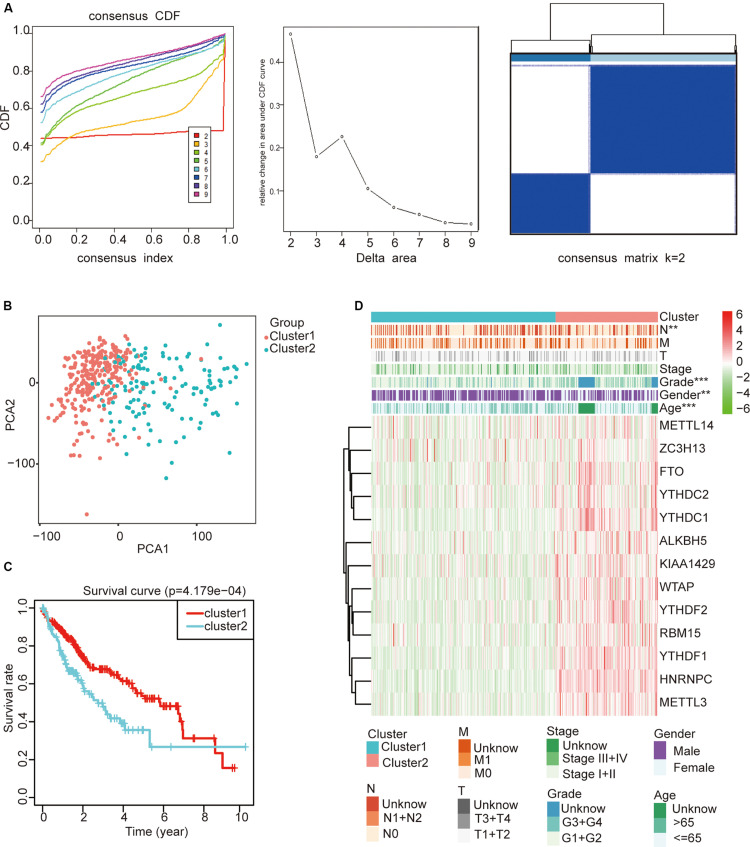
The role of subtypes based on m^6^A regulator expression profiling in hepatocellular carcinoma (HCC). **(A)** The relative change of the cumulative distribution function and the area under the curve of *k* = 2–9 for consensus clustering. This result shows that, when *K* = 2, the m^6^A regulators can well divide HCC into two types. **(B)** The results show that the subtypes identified based on the expression profile of the m6A modulator can well distinguish HCC into two clusters. **(C)** The comparison of survival curves between cluster 1 and cluster 2 subgroups. **(D)** The comparison of clinicopathological features between cluster 1 and cluster 2 subgroups (Wilcoxon test). **p < 0.01 and ***p < 0.001.

To study whether m^6^A regulators type HCC samples well, by inputting the expression profile of the m^6^A regulators, we performed a cluster analysis with the R package “ConsensusClusterPlus” (*k* = 2–9, Figure 4A). The results revealed that it was most appropriate to divide the patients into two subtypes (Figure 4A). These two subtypes were defined as cluster 1 and cluster 2 in order to further verify the accuracy of the two subtypes. We input all gene expression profiles and subtype information and used the R packages “limma” and “ggplot2” for the PCA of HCC. The PCA results also showed that the HCC sample could be well divided into two subtypes (Figure 4B). Moreover, a significantly shorter survival curve in the cluster 2 subgroup was observed (Figure 4C). Furthermore, the clinical characteristics of the two subtypes are shown in Table 2. These two subgroups were significantly correlated with the WHO grade, gender, age, and lymph node metastasis (*p* < 0.05) (Figure 4D). These findings further indicate that m^6^A regulators have a key role in HCC categories. However, the specific molecular differences or other effects between these two subtypes needed further research.

**TABLE 2:** The clinical features of hepatocellular carcinoma.

**Variables**	**Cluster 1 (*n* = 260)**	**Cluster 2 (*n* = 143)**	**High risk (*n* = 201)**	**Low risk (*n* = 202)**
**Age (years)**				
≤ 65	154	78	113	119
> 65	105	33	56	82
Unkonw	1	32	32	1
**Gender**				
Female	76	64	77	63
Male	184	79	124	193
**Grade**				
G1 + G2	183	49	86	146
G3 + G4	72	61	81	52
Unknown	5	33	34	4
**Tumor invasion (T)**				
T1 + T2	197	105	174	155
T3 + T4	60	38	54	44
Unknown	3	0	0	3
**Lymph node (N)**				
N0	177	100	147	130
N1 + N2	1	7	6	2
Unknown	83	36	48	70
**Metastasis (M)**				
M0	184	110	153	140
M1	3	4	4	3
Unknown	73	30	44	59
**Tumor stage**				
Stages I + II	188	95	137	146
Stages III + IV	56	40	53	43
Unknown	16	8	11	13

### A Risk Signature Built Using Three Selected m^6^A Regulators

**FIGURE 5:**
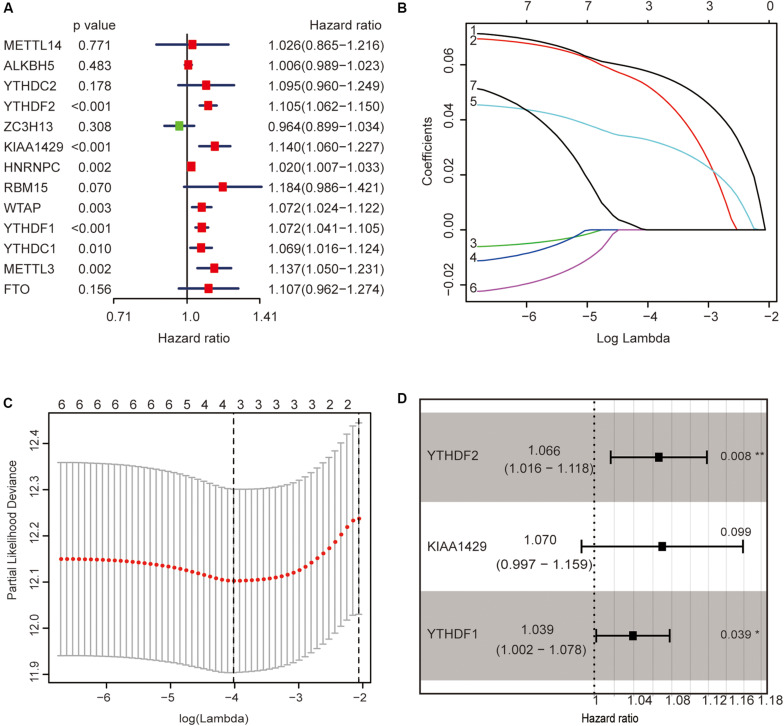
Identification of m^6^A regulators associated with hepatocellular carcinoma (HCC) prognosis. **(A)** The univariate Cox regression models identified seven m^6^A regulators associated with overall survival. **(B,C)** Three m^6^A regulators were identified by LASSO regression analysis. **(D)** Three m^6^A regulators were identified for constructing a prognostic model by multivariate Cox regression analysis. *p < 0.05 and **p < 0.01.

The previous results revealed that m^6^A regulators play an important role in HCC. In order to explore whether m^6^A regulators predict the survival prognosis of HCC patients, we combined the expression profile and the clinical data of m^6^A regulators for univariate Cox regression analysis. The results revealed that a total of seven genes (YTHDF2, KIAA1429, HNRNPC, WTAP, YTHDF1, YTHDC1, and METTL3) were significantly associated with survival prognosis (*p* < 0.05, Figure 5A). The hazard ratio values of these seven genes were all more than 1 (Figure 5A), indicating that they may be negative prognostic factors for HCC patients.

**FIGURE 6:**
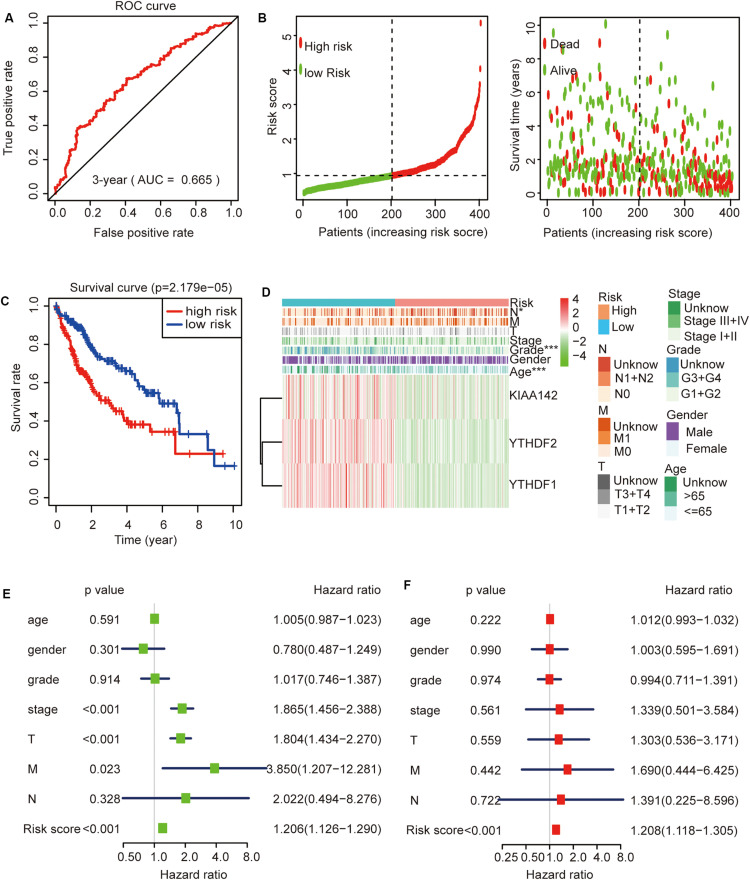
The role of this risk signature in hepatocellular carcinoma (HCC). **(A)** Receiver operating characteristic curve analysis predicts the accuracy of the 3-year survival of risk signature in HCC. **(B)** The risk score analysis of this risk signature in HCC. **(C)** The comparison of survival curves between high- and low-risk groups. **(D)** The comparison of clinicopathological features between high- and low-risk groups (Wilcoxon test). **(E)** The association between clinicopathological factors (including the risk score) and overall survival by univariate Cox regression analyses. **(F)** The association between clinicopathological factors (including the risk score) and overall survival by multivariate Cox regression analyses. *p < 0.05 and ***p < 0.001.

Then, we further analyzed these seven genes through LASSO regression analysis, and the results showed that three m^6^A regulators (YTHDF1, YTHDF2, and KIAA1429) might be able to construct a prognostic model (Figures 5B,C). A multivariate Cox regression analysis was used to construct a risk signature based on the expression of these three genes (Figure 5D). The univariate and multivariate Cox regression results are shown in Table 3. Risk score = 0.038 × expression of YTHDF1 + 0.064 × expression of YTHDF2 + 0.067 × expression of KIAA1429. The patients were divided into high-risk and low-risk groups by the median risk score (0.939), which served as the cutoff value. The model constructed with the risk signature showed that the AUC values of the time–ROC curve for 3-year overall survival (OS) was 0.665 (Figure 6A). As the risk score increased, the mortality rate increased gradually (Figure 6B). OS in the high-risk group was significantly shorter than in the low-risk group (*p* < 0.05, Figure 6C). The clinical characteristics of the high- and low-risk groups are shown in Table 2. The high- and low-risk groups were found to correlate significantly with age, grade, and lymph node metastasis in HCC (*p* < 0.05, Figure 6D). To further assess whether risk score can be used as an independent prognostic indicator, we performed univariate Cox and multivariate Cox regression analyses on the risk score. By univariate analysis, we found that the risk score, WHO grades, and TNM stages were all correlated with the OS (*P* < 0.001) (Figure 6E). Including these factors into the multivariate Cox regression, the risk score remained significantly associated with the OS (*p* < 0.001) (Figure 6F). All the results suggest that the prognostic survival models based on these three genes are useful for prognosis in HCC patients. The expression level of these three genes can be used as independent prognostic factors for HCC in the clinic.

**TABLE 3:** Univariate and multivariate Cox regression analyses of three m^6^A regulators in hepatocellular carcinoma.

**Variables**	**Univariate analysis**		**Multivariate analysis**		
	**Hazard ratio (HR) (95% CI)**	***P-*value**	**Coefficient**	**HR (95% CI)**	***P-*value**
*YTHDF2*	1.105 (1.062–1.150)	<0.001	0.064	1.066 (1.016–1.118)	0.008
*YTHDF1*	1.072 (1.041–1.105)	<0.001	0.038	1.039 (1.002–1.078)	0.039
*KIAA1429*	1.140 (1.060–1.227)	<0.001	0.067	1.070 (0.997–1.159)	0.099

### Construction of a Prognostic Nomogram

**FIGURE 7:**
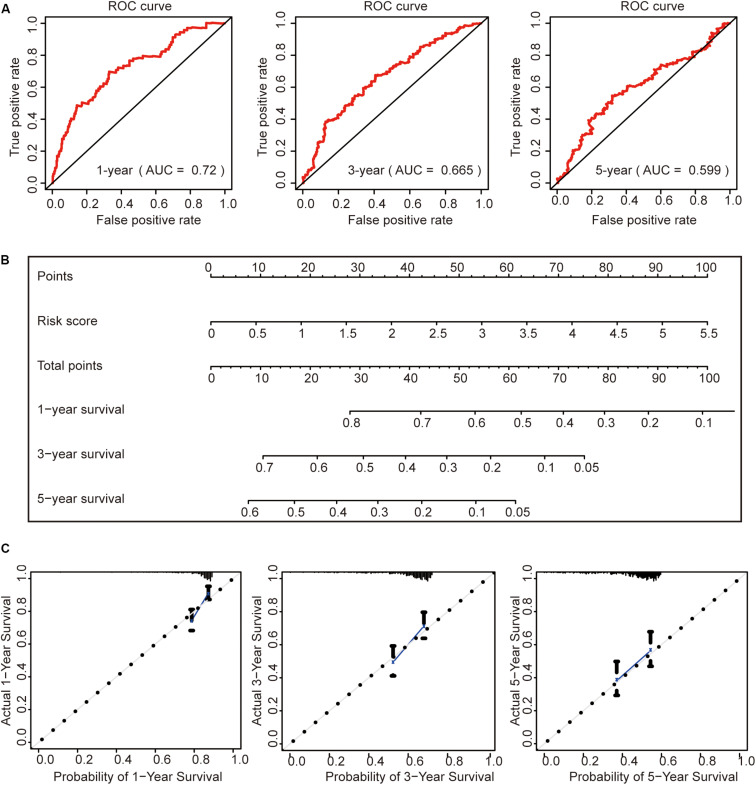
Construction of a prognostic model. **(A)** Receiver operating characteristic curve analysis of the ability of this risk signature to predict hepatocellular carcinoma (HCC) 1-, 3-, and 5-year survival status. **(B)** The construction of the nomogram was based on the risk score in HCC. **(C)** The calibration plot for internal validation of the nomogram.

To further evaluate this risk signature, we used the ROC curve to evaluate the model to predict the survival status of HCC for 1, 3, and 5 years, respectively. The results showed that the AUC value for 1 year is 0.72, the AUC value for 3 years is 0.665, and the AUC value for 5 years is 0.599 (Figure 7A). This result shows that the risk signature has a good prognosis for 1 and 3 years, but for the 5-year survival status, the prediction is not so accurate. The reason may be that the number of HCC patients in the TCGA data set who survived more than 5 years is too small. It may be better to add more samples for analysis.

Then, we constructed a nomogram to predict OS in patients with HCC based on risk scores (Figure 7B). The calibration plots showed that the performance of the nomogram was best in predicting 1-, 3-, and 5-year OS (Figure 7C).

**FIGURE 8:**
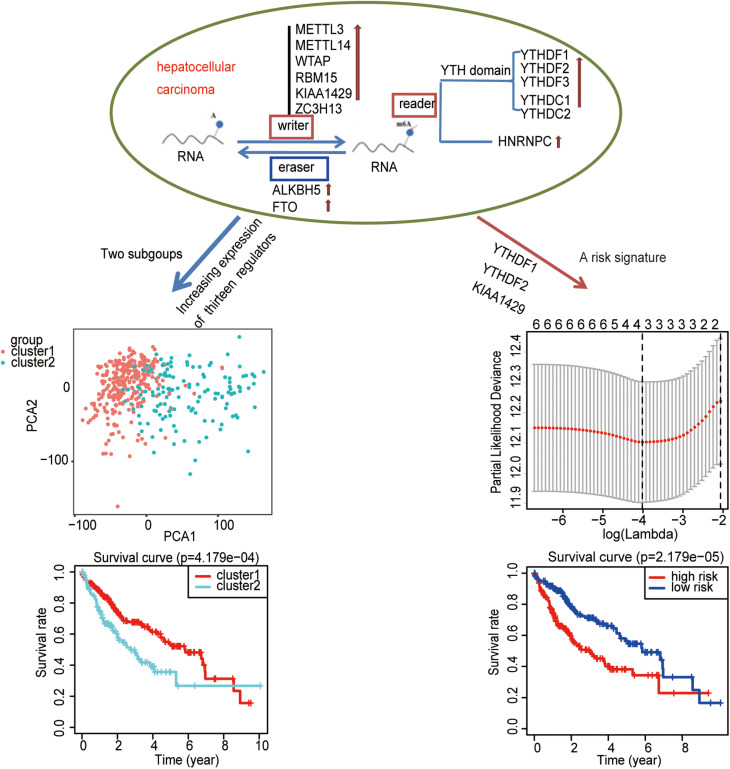
Summary of the potential prognostic value of m^6^A regulators in hepatocellular carcinoma.

Consequently, an independent prognostic risk signature was built based on three m^6^A regulators (YTHDF1, YTHDF2, and KIAA1429) in HCC (Figure 8).

## Discussion

Accumulating evidence shows that the m^6^A modification was observed in diverse cancers, which is important for cancer stem cells self-renewal, cancer cell proliferation, and radiotherapy or chemotherapy resistance (Pan et al., 2018). The formation of m^6^A is catalyzed by the prominent “writer” proteins (Duan et al., 2019). The downstream cellular functions of m^6^A rely on its “readers” (Wang et al., 2015; Kretschmer et al., 2018). In addition, HNRNPC is considered as an “m^6^A switch” to improve the accessibility of RNA binding proteins (Liu et al., 2015). Some reports show that METTL14 is supposed to be an oncogene in acute myeloid leukemia (Weng et al., 2018). WTAP also acts as an oncogene for the development of malignant tumors and a target for immunotherapy of cancer patients (Xie et al., 2019). KIAA1429 acts as an oncogenic factor in breast cancer and contributes to liver cancer progression (Lan et al., 2019; Qian et al., 2019).

Currently, increasing evidence indicates that m^6^A regulators are involved in the progression of HCC (Ma et al., 2017; Yang et al., 2017). The “writer” METTL3 contributes to HCC progression by repressing SOCS2 expression (Chen et al., 2018). The “writers” METTL14 acts as an adverse prognosis factor for HCC by promoting miR126 processing (Ma et al., 2017). KIAA1429 is involved in liver cancer progression and regulates the invasion of HCC by altering the m^6^A modification of ID2 and GATA3 (Qian et al., 2019; Cheng et al., 2019). The “reader” YTHDF2 was closely associated with the malignancy of HCC modulated by MiR145 (Yang et al., 2017). Our results are consistent with these reports. All m^6^A regulators, except ZC3H13, are highly expressed in HCC, indicating that m^6^A regulators have key roles in HCC. The PCA results show that m^6^A regulators can divide hepatocellular carcinoma patients into two types well, and two clustering subgroups have significant differences in WHO grade, gender, age, and lymph node metastasis. All these results suggest that m^6^A regulators may be a useful diagnostic classification tool for HCC. However, we only explore the relevance of these two types and clinical features. More detailed studies of m^6^A regulatory factors in the diagnostic classification of HCC are needed.

There is an important question of whether the m^6^A regulator expression level can act as a prognostic marker in HCC. Li et al. show that KIAA1429, METTL3, and HNRNPC are highly expressed in HCC tissues, while METTL14, ZC3H13, YTHDC1, YTHDC2, and FTO expressions are lower than those in normal tissues. A three-gene (CSAD, GOT2, and SOCS2) signature regulated by METTL14 is efficient for the prognostication of HCC (Li et al., 2020), which suggests that m^6^A regulators have a clinical prognostic impact in HCC. In our present study, we get similar results that the m^6^A regulator expression levels are essential for hepatocellular carcinoma prognosis. Differently, in our study, we derive the HCC prognostic signature from the expression of three m^6^A regulators (YTHDF1, YTHDF2, and KIAA1429). As we have observed, the three-gene signature generated by risk score can stratify the OS for HCC patients. In our results, the expression of all m^6^A regulators, except for ZC3H13, is higher in the tumor samples than in the adjacent normal tissue. Inconsistent results may result from different sample amounts and sources. More samples are used in our study than in their research, and all our study data of 407 samples are from the TCGA database, while 64 of 307 patients included in their report are from the GSE116174 dataset (others are from the TCGA database). Moreover, that report focuses on studying the function of METTL14 and establishing a METTL14-regulated three-gene (CSAD, GOT2, and SOCS2) signature and nomogram to predict the OS of HCC. However, in our study, the HCC prognostic signature derives from directly using three m^6^A regulators (YTHDF1, YTHDF2, and KIAA1429). The three regulators are considered to be useful markers for the diagnosis and the treatment of HCC patients in the clinic. Because the signature is generated based on the expression level of m^6^A regulators which do not involve the downstream target genes, additional trials are needed to find the target genes and the signaling pathways of these three regulators. That should be a good strategy to treat HCC by targeting YTHDF1, YTHDF2, and KIAA1429 combined with targeting their downstream genes.

In our results, a very surprising one is that ZC3H13 expression has no difference between tumor samples and adjacent tissue samples. In addition, ZC3H13 is not correlated with ALKBH5, KIAA1429, and YTHDF1. The previous report shows that the expression of ZC3H13 is lower than those in normal tissues (Li et al., 2020). ZC3H13 is a classical CCCH zinc finger protein localized in human chromosome 13q14.139 (Ouna et al., 2012). As an m^6^A methylation writer, the role of ZC3H13 in tumors is controversial. A report shows that ZC3H13 serves as a tumor suppressor protein in colon carcinoma and colorectal cancer by regulating the Ras-ERK signaling pathway (Zhu et al., 2019). Other reports consider it as an oncogenic protein by binding with K-ras and activating the NF-κB signal (Knuckles et al., 2018). The controversial roles of ZC3H13 in tumors give us a clue that the essentiality and the functions of m^6^A RNA methylation in tumors are complicated, and further studies are needed to focus on its prognostic value in HCC.

Another interesting result is that RNA binding protein HNRNPC expression is elevated in HCC. This is consistent with the previous report (Li et al., 2020). The essentiality of HNRNPC in tumors is not clear. Certain studies show that HNRNPC promotes cell proliferation, apoptosis, and tumor growth (Kleemann et al., 2018; Wu et al., 2018). In addition, a high expression of HNRNPC has a poor prognosis and may act as a candidate biomarker for chemoresistance in gastric cancer (Huang et al., 2016). Besides that, HNRNPC also acts as a dengue virus NS1-interacting protein and plays an important role during the replication of the hepatitis C virus and hepatitis delta virus (Noisakran et al., 2008; Casaca et al., 2011). Our results imply that HNRNPC is a candidate biomarker for HCC. More work is needed to verify the relevant regulatory pathways.

Among 13 m^6^A RNA methylation regulators, the m^6^A methylation writer VIRMA (KIAA1429) has the most obvious mutation in HCC. VIRMA is identified as the component associated with WTAP in mammalian cells and involved in the regulation of m^6^A methylation events in 3’UTR and near the stop codon (Lobo et al., 2019). Certain studies show that KIAA1429 contributes to liver cancer progression through N6-methyladenosine-dependent post-transcriptional modification of GATA3 and regulates the migration and the invasion of HCC by altering the m^6^A modification of ID2 mRNA (Cheng et al., 2019; Qian et al., 2019). It is necessary to study the roles of obvious mutation of VIRMA in HCC occurrence and progression.

## Conclusion

In conclusion, a high expression of m^6^A regulators implies that dysregulated m^6^A play important roles in HCC. Furthermore, two clustering subgroups indicate that m^6^A RNA methylation plays essential roles in the prognosis and the clinicopathological features of HCC. In addition, a prognostic risk signature with three selected m^6^A RNA methylation regulators gives us a clue that m^6^A RNA methylation regulators are potentially useful for prognostic stratification and targeting treatment in HCC.

## Data Availability Statement

The RNA-seq transcriptome and clinicopathological datas of 407 HCC samples and 58 adjacent normal tissue samples were obtained from TCGA (https://portal.gdc.cancer.gov/).

## Author Contributions

WL and MT designed the study. WL and FX performed the analysis and drafted the manuscript. WL, MT, CZ, DL, and FX contributed to the editing of the manuscript. All authors contributed to the article and approved the submitted version.

## Conflict of Interest

The authors declare that the research was conducted in the absence of any commercial or financial relationships that could be construed as a potential conflict of interest.

## Footnotes

1 https://portal.gdc.cancer.gov/

2 http://www.cbioportal.org/

3 http://www.bioconductor.org/

4 http://www.cbioportal.org/
